# Phasin PhaP1 is involved in polyhydroxybutyrate granules morphology and in controlling early biopolymer accumulation in *Azospirillum brasilense* Sp7

**DOI:** 10.1186/s13568-019-0876-4

**Published:** 2019-09-25

**Authors:** María de los Angeles Martínez-Martínez, Bertha González-Pedrajo, Georges Dreyfus, Lucía Soto-Urzúa, Luis Javier Martínez-Morales

**Affiliations:** 1 Centro de Investigaciones en Ciencias Microbiológicas, Instituto de Ciencias, Benemérita Universidad Autónoma de Puebla, Puebla, Mexico; 2 Departamento de Genética Molecular, Instituto de Fisiología Celular, Universidad Nacional Autónoma de México, Mexico City, Mexico

**Keywords:** *Azospirillum brasilense* Sp7, Phasins, C:N ratio, Oxygen stress, PHB accumulation

## Abstract

Phasins are amphiphilic proteins involved in the regulation of the number and size of polyhydroxybutyrate (PHB) granules. The plant growth promoting bacterium *Azospirillum brasilense* Sp7 accumulates high quantities of bioplastic PHB as carbon and energy source. By analyzing the genome, we identified six genes that code for proteins with a Phasin_2 domain. To understand the role of *A. brasilense* Sp7 PhaP1 (PhaP1_Abs_) on PHB synthesis, the *phaP1* gene (AMK58_RS17065) was deleted. The morphology of the PHB granules was analyzed by transmission electron microscopy (TEM) and the PHB produced was quantified under three different C:N ratios in cultures subjected to null or low-oxygen transfer. The results showed that PhaP1_Abs_ is involved in PHB granules morphology and in controlling early biopolymer accumulation. Using RT-PCR it was found that phasin genes, except *phaP4*, are transcribed in accordance with the C:N ratio used for the growth of *A. brasilense. phaP1*, *phaP2* and *phaP3* genes were able to respond to the growth conditions tested. This study reports the first analysis of a phasin protein in *A. brasilense* Sp7.

## Introduction

Since it was reported for the first time in 1926 (Lemoigne 1926), polyhydroxybutyrate (PHB) has been the best studied bacterial biopolymer. PHB exhibits similar characteristics with respect to petrochemical plastics in addition to its biodegradability and biocompatibility properties. These characteristics enable PHB convenient to reduce the use of petrochemical plastic derivatives (Madison and Huisman 1999; Anjum et al. 2016). Besides the significant advantages of PHB, it is currently not used in large quantities because of the high production costs (US$ 5.5/kg) (Brigham and Riedel 2018; Pavan et al. 2019), mainly caused by the expensive carbon sources and the ineffective recovery process. A solution for improve the large-scale production of PHB could be the use of economic carbon substrates for PHB production such as food wastes (Brigham and Riedel 2018). Nonetheless, usage of cheaper carbon source combined with optimized processes for PHA accumulation and extraction could reduce the cost of biopolymer production and this could allow to address the large-scale production challenge. Since the demand in the health industry, the making of renewable materials and the advances in technologies, will make the market increase at a rate of 6.3%, reaching 119.15 million dollars by 2025 (Global Polyhydroxyalkanoate (PHA) Market Analysis & Trends—Industry Forecast to 2025).

More than 300 microorganisms have been reported to be PHB-producers (Koller et al. 2010; Alarfaj et al. 2015). PHB-producing microorganisms synthesize PHB by three enzymatic reactions. β-ketothiolase (PhaA) condenses two acetyl-CoA into acetoacetyl-CoA, then acetoacetyl-CoA is reduced to 3-hydroxybutyril-CoA by an acetoacetyl-CoA reductase (PhaB) and finally, 3-hydroxybutyril-CoA monomers are polymerized to PHB by a PHB synthase (PhaC) (Madison and Huisman 1999; Sagong et al. 2018).

Once synthesized, PHB is packaged into granules named carbonosomes which contain PHB covered by granule associated proteins (GAP’s) (Steinbüchel et al. 1995; Jendrossek 2009; Pfeiffer and Jendrossek 2012). Four main GAP’s have been reported: PHB synthases (PhaC), PHB depolymerases (PhaZ), regulators (PhaR or PhaM) and phasins (PhaP) (Steinbüchel et al. 1995; Tirapelle et al. 2013). Synthases and depolymerases are involved in PHB accumulation and utilization, respectively (Juengert et al. 2018; Mezzolla et al. 2018). Phasins are small amphiphilic proteins that coat and stabilize PHB in the cytoplasm (Sznajder et al. 2015) and therefore, phasins are involved in regulate the number and size of carbonosomes (Pfeiffer and Jendrossek 2012; Hauf et al. 2015). Regulator proteins (PhaR) control phasin synthesis (Maehara et al. 2002; Pötter et al. 2002; York et al. 2002) and possibly granule localization (PhaM) (Pfeiffer et al. 2011; Pfeiffer and Jendrossek 2014).

It has been demonstrated that phasins are the main GAP’s (Steinbüchel et al. 1995: Sznajder et al. 2015). Studies on phasins have suggested possible interactions with PHB polymerases and PHB depolymerases depending on the PHB-producing microorganism (York et al. 2001; Fukui et al. 2001; Handrick et al. 2004a, b; Tian et al. 2005; Ushimaru et al. 2014). In *Aeromonas hydrophila* the PHB content increased twofold when the only PhaP was overexpressed (Ushimaru et al. 2014), also changes in the monomer composition of poly(3-hydroxybutyrate-*co*-3-hydroxyhexanoate) [P(HB-*co*-HHx)] copolyester were documented (Tian et al. 2005). It has also been shown that PHB is easily degraded in the presence of PhaP in *Rhodospirillum rubrum* (Handrick et al. 2004a, b; Kuchta et al. 2007).

PHB synthesis has been poorly studied in the α-proteobacterium *A. brasilense* Sp7. This bacterium accumulates up to 70% of its dry-cell weight (DCW) as PHB. The PHB accumulated energizes metabolic processes that favor bacterial survival under stressful environments (Tal and Okon 1985; Itzigsohn et al. 1995). PHB accumulation in *A. brasilense* Sp7 requires an excess in carbon and limited nitrogen sources (Tal and Okon 1985; Itzigsohn et al. 1995). Malic acid and ammonium chloride are the best carbon and nitrogen sources to improve PHB accumulation in *A. brasilense* Sp7 (Itzigsohn et al. 1995). Biochemical analyses of the PHB synthase and PHB depolymerase in *A. brasilense* have been carried out (Tal et al. 1990; Kadouri et al. 2002, 2003). However, there are no reports dealing with phasin proteins.

Our goal in this study was to analyze the function of the PhaP1_Abs_ on the morphology of PHB granules and PHB accumulation using carbon:nitrogen (C:N) ratios of 30:1, 60:1 and 90:1, in cultures subjected to null or low-oxygen transfer. By means of bioinformatics analyses we found six genes that code for proteins containing a Phasin_2 domain (PF09361). In this work we deleted *phaP1* gene and the resulting mutant was characterized. The results showed that PhaP1_Abs_ controls the morphology of the PHB granules and is involved in the control of early PHB synthesis in some of the growth conditions evaluated.

## Materials and methods

### Bacterial strains, plasmids and growth conditions

Bacteria used in this study are listed in Table 1. *Escherichia coli* strains were grown in Luria Bertani (LB) broth supplemented with the appropriate antibiotics at 37 °C and 150 rpm. LB composition per liter: yeast extract 5 g, casein peptone 10 g and NaCl 10 g. For *A. brasilense*, a minimal medium supplemented with malic acid and ammonium chloride as carbon and nitrogen sources, was used. Minimal medium composition per liter: Malic acid 4.355 g, NH_4_Cl 0.2 g, K_2_HPO_4_ 1.67 g, KH_2_PO_4_ 0.87 g, MgSO_4_ 0.29 g, NaCl 0.48 g, CaCl_2_ 0.07 g, FeCl_3_·6H_2_O 0.01 g, Na_2_MoO_4_·2H_2_O 0.025 g and 10 mL of trace element solution (containing MnSO_4_·H_2_O 250 mg, ZnSO_4_·7H_2_O 70 mg, CoSO_4_·7H_2_O 14 mg, CuSO_4_·5H_2_O 12.5 mg and H_3_BO_3_ 3 mg per liter). *A. brasilense* Sp7 strains were cultured, with the appropriated antibiotics, at 32 °C and 110 rpm. All incubations were carried out in a 25 mm amplitude orbital shaker (INO-650M SEV^®^).

**Table 1:** Strains and plasmids used in this study

Bacteria	Characteristics	Reference
*Azospirillum brasilense* Sp7	Wild type strain	Tarrand et al. (1978)
*A. brasilense* Δ*phaP1*	*A. brasilense* Sp7 deleted in *phaP1* gene.	This study
*A. brasilense* PhaP1^+++^	*A. brasilense* Δ*phaP1* containing pMBA-12	This study
*E. coli* S17-1	*Sm*r, *rec*A, *thi*, *pro*, *hsd*R-M+ RP4:2-*Tc*:*Mu*:*Km* Tn7, λ*pir*	Simon et al. (1983)

Plasmids	Characteristics	References
pCR2.1TOPO	Source of Km resistance cassette	Invitrogen
pGLO	Source of Ara promoter	BioRad
pMMB206	Expression vector	Morales et al. (1991)
pSUP202	Suicide vector	Roschanski and Strauch (2011)
pSUPAB	pSUP202 containing upstream and downstream *phaP1* flanking regions.	This study
pSUPAKB	pSUPAB containing Km resistance cassette of pCR2.1TOPO	This study
pMBA-2	Expression vector pMMB206 derivative containing Ara promoter	This study
pMBA-12	pMBA-2 with *phaP1*	This study

### Bioinformatics analyses

Proteins containing a Phasin_2 domain (PF09361) in the *A. brasilense* Sp7 genome were searched on National Center for Biotechnology Information (NCBI) (Geer et al. 2009), Simple Modular Architecture Research Tool (SMART) (Letunic and Bork 2017) and PFAM databases (Finn et al. 2015). Secondary structures were predicted using the PSI-blast based secondary structure PREDiction (PSIPRED) software (Buchan et al. 2013) and Protein Homology/AnalogY Recognition Engine (Phyre2) server (Kelley et al. 2015). Three-dimensional structures of putative phasins were predicted by using the Iterative Threading ASSEmbly Refinement (I-TASSER) (Roy et al. 2010; Yang and Zhang 2015; Zhang et al. 2017) and SWISS-MODEL (Waterhouse et al. 2018) servers. The obtained three-dimensional structures were evaluated by Qualitative Model Energy ANalysis (QMEAN) (Benkert et al. 2011) and Ramachandran plots at SWISS-MODEL Structure Assessment server. Alignments were done using Clustal X software (Larkin et al. 2007). Identity and similarity percentages were calculated on the Sequence Manipulation Suite (Stothard 2000). For the phylogenetic analyses, 18 amino acid sequences of phasins from main PHB-producing microorganisms were aligned using Clustal X software (Larkin et al. 2007). The phylogenetic tree was inferred using the Neighbor-Joining method and the evolutionary distances were computed using the Poisson correction method at the Molecular Evolutionary Genetics Analysis software version 7.0 (MEGA 7.0) (Kumar et al. 2016). The number of replicates were 500. Amino acid sequences of *Ralstonia eutropha* phasins were used as outgroup for the tree.

### DNA manipulation

DNA was obtained by the phenol:chloroform method as previously reported (Cheng and Jiang 2006). Briefly, bacterial cells were harvested by centrifugation (8000×*g* for 5 min), the supernatant was removed, and the pellet washed in Sodium chloride–Tris–EDTA (STE) buffer twice. Cells were recovered and resuspended in Tris–EDTA (TE) buffer. Tris-saturated phenol (pH 8) was added, and the samples were mixed by vortexing and centrifuged as above. Phenol traces and debris were cleaned by three chloroform washes and the aqueous phase containing nucleic acids was recovered. RNA was eliminated by adding RNase H (10 mg/mL) to the aqueous phase and incubating for 10 min at 37 °C followed by purification with phenol:chloroform (Invitrogen). Pure DNA was contained in the aqueous phase. Plasmid DNA was isolated by the miniprep technique (Sambrook et al. 1989). Cloning procedures such as PCR amplifications, digestions and ligations were performed according to the instructions provided by the manufacturer (ThermoScientific).

### In frame deletion of the *phaP1* gene in *A. brasilense* Sp7

Upstream and downstream regions of the *phaP1* gene (AMK58_RS17065) were PCR amplified using the primers *phaP1*FA*Pst*I/*phaP1*RA*Xma*I (upstream) and *phaP1*FB*Xma*I/*phaP1*RB*Eco*RI (downstream) (Table 2). Primers were designed to contain *Pst*I/*Xma*I and *Xma*I/*Eco*RI restriction sites. PCR conditions consisted of an initial denaturalization step at 94 °C for 5 min, followed by 30 cycles of denaturing at 94 °C for 45 s, annealing at 58 °C for 45 s and extension at 72 °C for 1 min as suggested by the manufacturer (ThermoScientific). Finally, an additional extension of 5 min at 72 °C was carried out. Both PCR fragments were digested with the specified endonucleases and cloned into *Pst*I/*Eco*RI sites of the suicide vector pSUP202 generating the plasmid pSUPAB. Then, kanamycin resistance gene from pCR2.1TOPO vector was cloned into *Xma*I sites of pSUPAB. The resulting suicide vector, pSUPAKB, was mobilized into *A. brasilense* Sp7 using *E. coli* S17-1 as a donor strain as previously reported (Mishra et al. 2011). The *A. brasilense phaP1* deleted mutant strain (named *A. brasilense* Δ*phaP1*) was selected on minimal medium supplemented with kanamycin (50 µg/mL) and ampicillin (100 μg/mL).

**Table 2:** Primers used in this study (restriction sites are underlined)

Primer	Sequence (5′ → 3′)
*pha*P1FA*Pst*I	gatactgcagcgccaacctgatcgagcata
*pha*P1RA*Xma*I	tatccccgggcttggccatggttctcaccc
*pha*P1FB*Xma*I	gatacccgggaagaagtaagcgtctctcccg
*pha*P1RB*Eco*RI	ctatgaattcaaggaactgctggtcatctc
*pha*P1-F*Mfe*I	ctagcaattgcatggccaagcagaccggtaa
*pha*P1-R*Sma*I	atacccgggttacttcttggcgatgcgggc
RT-16 s rRNA-F	ctgaacaaccagcgcatcga
RT-16 s rRNA-r	aagtcctcgcgcttcacg
RT-*pha*P1-F	gccgagatcgtccgttcct
RT-*pha*P1-R	gcgaccttgaccagttcgg
RT-*pha*P2-F	cccagtcctcgctggagaag
RT-*pha*P2-R	gtctcgaagctcgccttggc
RT-*pha*P3-F	agctcctgaagatctcctccga
RT-*pha*P3-R	cctcacgggtcagctcctcg
RT-*pha*P4-F	cctgtccgcacaagccca
RT-*pha*P4-R	ccattcgcgcatgatggac
RT-*pha*P5-F	gccaatccggagttcgtcc
RT-*pha*P5-R	ccctgcatcagggtcatcca
RT-*pha*P6-F	cgtcaaccagatgcagaccgtc
RT-*pha*P6-R	cggagcagccttcggagc

### Complementation of the *A. brasilense* Δ*phaP1* strain

The complementation of the *A. brasilense* Δ*phaP1* was done in two steps. First, the pMBA-2 vector was generated by cloning of the arabinose promoter and the *araC* gene from pGLO into *ApaI*/*EcoRI* sites of the expression vector pMMB206. Second, the *phaP1* gene was amplified by PCR using the primers *phaP1*-F*Mfe*I/*phaP1*-R*Sma*I and wild type total DNA as a template. The PCR product was digested and ligated into *Eco*RI/*Sma*I restriction sites of pMBA-2. The resulting pMBA-12 plasmid was mobilized into *A. brasilense* Δ*phaP1* using *E. coli* S17-1 as a donor strain. The complemented mutant (named *A. brasilense* PhaP1^+++^) was selected on minimal medium supplemented with chloramphenicol (10 µg/mL), kanamycin (50 µg/mL) and ampicillin (100 µg/mL). Induction of the arabinose promoter for PhaP1_Abs_ expression in pMBA-12 vector was carried out with 0.1% arabinose (Sigma-Aldrich).

### Growth curves of *A. brasilense* strains

Flasks containing 50 mL of minimal medium supplemented with malic acid and ammonium chloride at C:N ratios of 30:1, 60:1 and 90:1 were inoculated with the *A. brasilense* strains to an optical density at 600 nm (OD600) of 0.01, then the cultures were grown at 32 °C at 110 rpm. The OD600 was determined every 3 h during 24 h.

### PHB quantitation

Strains of *A. brasilense* were grown on minimal medium supplemented with malic acid and ammonium chloride at C:N ratios of 30:1, 60:1 and 90:1 in flasks subjected to null or low-oxygen transfer. To generate null-oxygen transfer condition, unbaffled Erlenmeyer flasks of 125 mL of capacity were filled with 25 mL of minimal medium and capped with a number 5 rubber stopper, which keep off from the atmospheric oxygen transfer throughout the assay. To generate low-oxygen transfer condition, unbaffled Erlenmeyer flasks of 125 mL of capacity were filled with 25 mL of minimal medium and capped with a 5 g cotton stopper, which keep off from the atmospheric oxygen transfer throughout the assay. The flasks containing the minimal medium were inoculated with an overnight culture to an OD600 of 0.01 and grown at 32 °C and 110 rpm. For each strain, four flasks with 25 mL of minimal medium were inoculated with the appropriated strain. One flask was uncovered at 24 h to determine PHB production, another flask was uncovered at 48 h and so on. This led us to maintain the oxygen conditions throughout the study. Samples of *A. brasilense* strains (10 mL) were taken at 24, 48, 72 and 96 h and harvested by centrifugation (6000×*g* for 5 min). Cells were washed with TE buffer twice, weighed and dried at 60 °C until weight was constant. PHB was extracted as reported elsewhere (Hahn et al. 1994) and quantified spectrophotometrically (Law and Slepecky 1961). Data were correlated with a commercial standard of PHB (Sigma-Aldrich). Determinations were carried out in triplicate. Results are shown as mean ± standard deviation of % PHB/DCW. Statistical analysis was performed in Statgraphics Centurion 18 software using One-way ANalysis Of VAriance (ANOVA) at 95% confidence level.

### Transmission electron microscopy (TEM)

Samples of *A. brasilense* strains were grown at C:N ratios of 30:1, and 90:1, harvested (6000×*g* for 5 min) and washed with TE buffer twice. Cells were fixed in 2.5% (v/v) glutaraldehyde for 3 h, postfixed in 1% (w/v) OsO_4_ for 2 h and rinsed 3 times in 50 mM of sodium cacodylate buffer (pH 7.2). Bacteria were dehydrated with a graded ethanol series and embedded in a low-viscosity epoxy resin that was subsequently dried at 60 °C for 24 h. Ultra-thin sections were stained with uranyl acetate and examined on a Jeol JEM-1200 EX II transmission electron microscope.

### RT-PCR analysis

RNA was extracted from cultures of *A. brasilense* strains (wild type, mutant and complemented) grown on minimal medium at C:N ratios of 30:1 and 90:1. The extraction was performed using the Trizol reagent method according to the instructions provided (Sigma-Aldrich). A mix consisting of: 1 μg of pure RNA, *phaP1* to *phaP6* gene-specific primers (RT-*pha*P1-R, RT-*pha*P2-R, RT-*pha*P3-R, RT-*pha*P4-R, RT-*pha*P5-R and RT-*pha*P6-R) (Table 2), dNTP mix, RNase inhibitor, RevertAid reverse transcriptase and the RevertAid reverse transcriptase buffer, was used to produce the cDNA according to the recommendations of the manufacturer (ThermoScientific). For the PCR amplification of each putative phasin gene (internal region of ~ 120 nucleotides), a mix consisting of: cDNA (1 μL), dNTPs, gene specified pair of primers (RT-*pha*P1-F/RT-*pha*P1-R, RT-*pha*P2-F/RT-*pha*P2-R, RT-*pha*P3-F/RT-*pha*P3-R, RT-*pha*P4-F/RT-*pha*P4-R, RT-*pha*P5-F/RT-*pha*P5-R or RT-*pha*P6-F/RT-*pha*P6-R) (Table 2), Taq polymerase recombinant and Taq polymerase recombinant buffer, was utilized. PCR conditions consisted of an initial denaturalization step at 94 °C for 5 min, followed by 30 cycles of denaturing at 94 °C for 30 s, annealing at 58 °C for 30 s and extension at 72 °C for 1 min as suggested by the manufacturer (ThermoScientific). Finally, an additional extension of 5 min at 72 °C was carried out. 16S rRNA gene (AMK58_09830) was used as control. PCR products were analyzed by gel electrophoresis on 3% agarose gels and subsequent ethidium bromide staining. RT-PCR assays were repeated three times.

## Results

### *A. brasilense* Sp7 contains six putative *phaP* genes

In silico analyses of the *A. brasilense* Sp7 genome revealed six genes that code for proteins containing a Phasin_2 domain (PF09361). Two putative phasin genes are in plasmid 1: AMK58_RS17065 (Gene ID: 36110503) and AMK58_RS20955 (Gene ID: 36111302) and the other putative phasin genes are in the chromosome: AMK58_RS04265 (Gene ID: 36107916), AMK58_RS04270 (Gene ID: 36107917), AMK58_RS07520 (Gene ID: 36108566) and AMK58_RS13850 (Gene ID: 36109844) (Additional file 1). The putative phasin proteins were referred as PhaP1_Abs_ to PhaP6_Abs_ according to the e-value (Table 3) presented with respect to the Phasin_2 domain. The size of the PhaP proteins ranges from 15 to 29 kDa. Analysis of the predicted secondary structure showed that phasins PhaP1_Abs_ to PhaP5_Abs_ possess a high α-helix content (85 to 95%) in contrast to PhaP6_Abs_ which shows a low percentage (14%) (Table 3).

**Table 3:** Characteristics of *A. brasilense* Sp7 phasin proteins

Phasin protein	Location	E-value^a^	Length^b^	Phasin_2 domain location	α-helix		MW (KDa)
					Percentage	Position^c^	
PhaP1_Abs_ (AMK58_RS17065)	ABSP7_p1: 722945–723373	4.56e^−15^	142	29–125	85	P8–E20 V28–A79 E85–I139	15.23
PhaP2_Abs_ (AMK58_RS04265)	Chr: 940913–941398	3.59e^−14^	161	46–142	94	K4–L96 I101–K158	16.98
PhaP3_Abs_ (AMK58_RS04270)	Chr: 941668–942204	3.89e^−12^	178	67–162	92	T2–K8 F17–L116 L121–I176	18.57
PhaP4_Abs_ (AMK58_RS07520)	Chr: 1633959–1634525	4.61e^−10^	188	79–174	95	E4–M128 V133–R185	20.37
PhaP5_Abs_ (AMK58_RS13850)	Chr: 2997626–2998141	6.20e^−03^	171	61–155	87	P11–A49 I55–L109 P115–Q169	18.34
PhaP6_Abs_ (AMK58_RS20955)	ABSP7_p1: 1595843–1596694	3.42e^−03^	283	25–111	14	A3–F12 E31–A35 T38–A57 A80–R83	28.84

^a^The E-value was obtained from the alignment of each phasin protein with the Phasin_2 domain

^b^Amino acids

^c^Amino acids

**Fig. 1:**
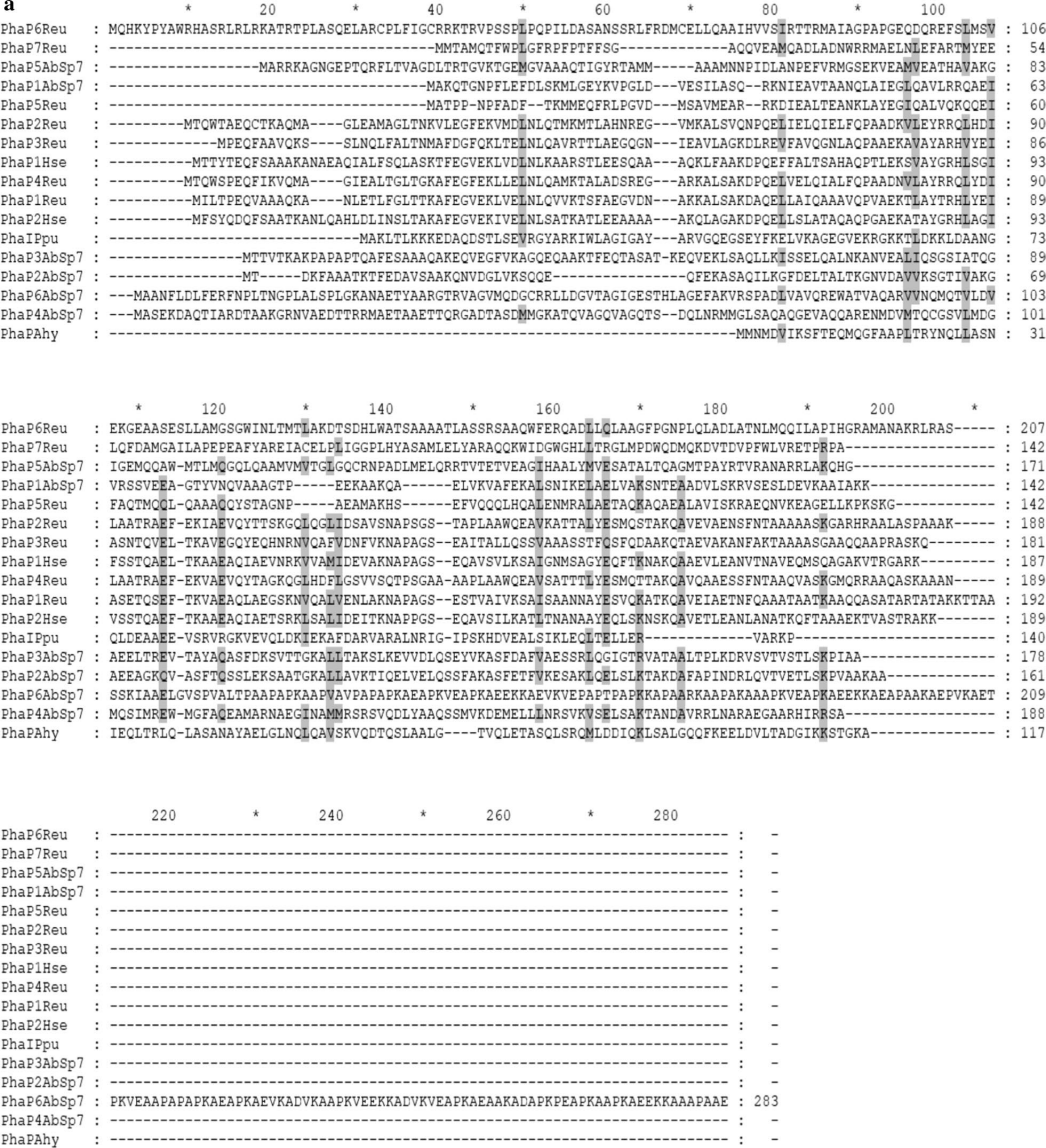

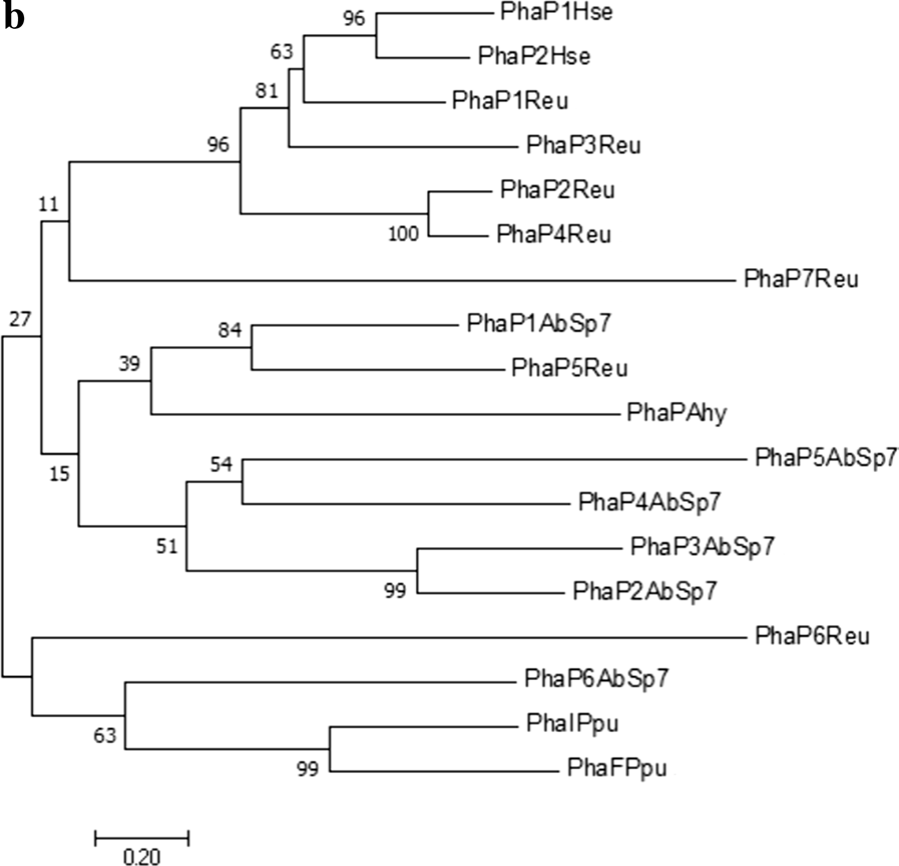
Phylogeny of *A. brasilense* Sp7 phasin proteins. **a** The amino acids sequences of phasin proteins of PHB-producing microorganisms were aligned to *A. brasilense* Sp7 proteins containing a phasin domain (PF09361). The conserved residues are highlighted. **b** A phylogenetic tree based on amino acid sequences of phasins was inferred using the Neighbor-Joining method. The percentage of replicate trees in which the associated taxa clustered together in the bootstrap test (500 replicates) are shown next to the branches. The evolutionary distances were computed using the Poisson correction method and are in the units of the number of amino acid substitutions per site. The analysis involved 18 amino acid sequences. All the positions containing gaps and missing data were eliminated. PhaP1AbSp7 to PhaP6AbSp7 denotes the *A. brasilense* Sp7 PhaP1 to PhaP6. PhaP1Reu to PhaP7Reu indicates the phasins PhaP1 to PhaP7 of *R. eutropha*. PhaIPpu and PhaFPpu are the phasins PhaI and PhaF, respectively, of *P. putida*. PhaP1Hse and PhaP2Hse are the *H. seropedicae* PhaP1 and PhaP2. PhaPAhy denotes the *A. hydrophila* PhaP

The phasin amino acid sequences from other PHB-producing microorganisms [*R. eutropha* (PhaP1 to PhaP7 and PhaM), *Pseudomonas putida* (PhaI and PhaF), *Aeromonas hydrophila* (PhaP), *Herbaspirillum seropedicae* (PhaP1 and PhaP2) and *Azotobacter* FA8 (PhaP)] were used for protein alignment with the *A. brasilense* Sp7 phasins (Fig. 1a). The results obtained showed that some *A. brasilense* Sp7 phasins are moderately conserved among phasins previously reported, with identities ranging from 3 to 33.7% and similarities from 6 to 55.8% (Additional file 2). The low identity and similarity percentages between the analyzed phasins, occurs by the diversity of aligned amino acid sequences of phasins belonging to the α, β, and γ-proteobacteria classes and does not pretend to identify a phasin domain. Moreover, the entire amino acid phasin sequences from the above-mentioned microorganisms were used to make a Neighbor-Joining phylogenetic tree (Fig. 1b). The putative PhaP2_Abs_, PhaP3_Abs_, PhaP4_Abs_ and PhaP5_Abs_ were clustered together on the 500 bootstrap tree. PhaP1_Abs_ maybe share a common ancestor with this clade. The putative PhaP6_Abs_ was clustered alone. A gene duplication event is suggested for PhaP2_Abs_ and PhaP3_Abs_. It is important to mention that was not possible enroot the phylogenetic tree due to the diversity of amino acid sequences of phasins used for tree construction.

**Fig. 2:**
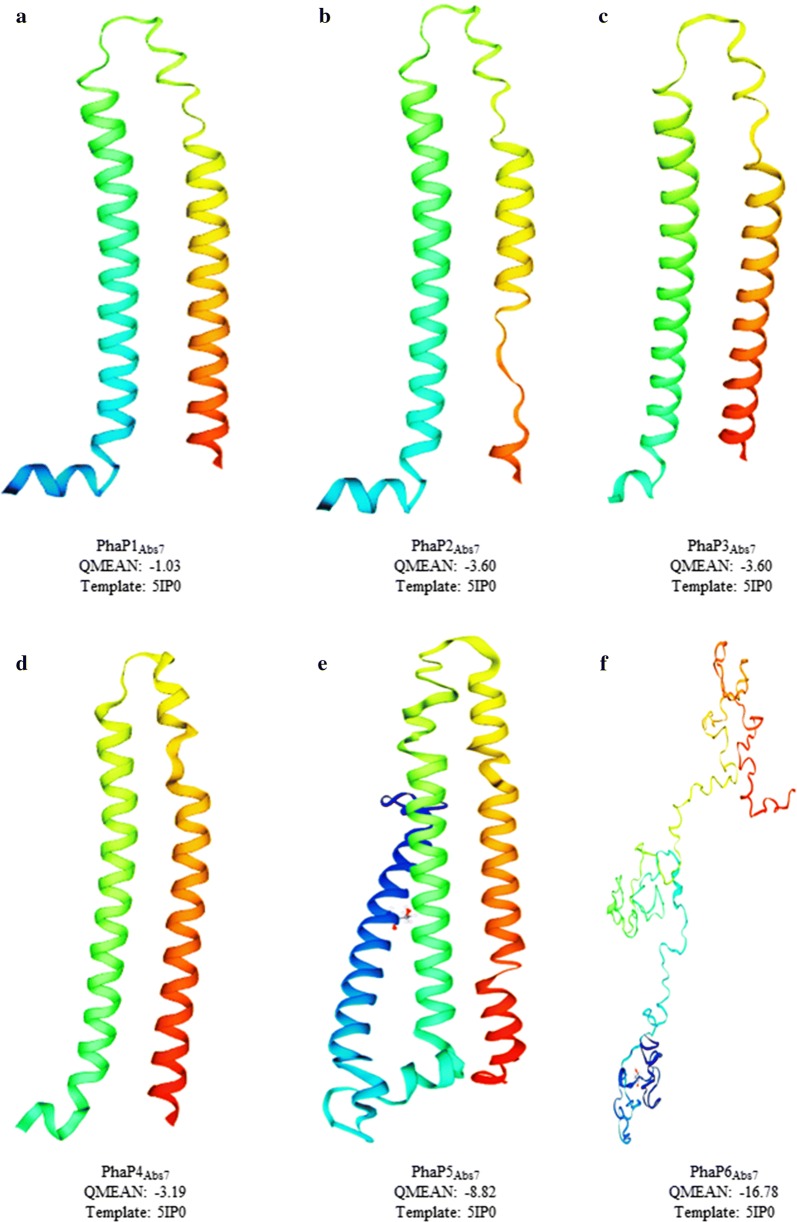
Predicted three-dimensional structures of *A. brasilense* Sp7 phasins. Structures were predicted using the I-TASSER and SWISS-MODEL servers. The crystal structure of PhaP from *A. hydrophila* (PDB number 5IP0) was used as template. For each *A. brasilense* Sp7 phasin, the QMEAN value was calculated and the Ramachandran plots were obtained (see Additional file 3). **a** PhaP1_Abs_, **b** PhaP2_Abs_, **c** PhaP3_Abs_, **d** PhaP4_Abs_, **e** PhaP5_Abs_ and **f** PhaP6_Abs_

Because of the non-existence of genetic, biochemical and structural information about phasin proteins of the most-well studied PHB-producing microorganisms, it was decided to analyze whether the putative *A. brasilense* Sp7 phasins share any structural homology with the crystal structure of PhaP_Ahy_ (*Aeromonas hydrophila*) (PDB number 5IP0) (Zhao et al. 2016). The PhaP_Ahy_ was used as template to predict the three-dimensional structures of the *A. brasilense* Sp7 phasins by the I-TASSER and SWISS-MODEL servers (Fig. 2 and Additional file 3). The results showed a good structural similarity for PhaP1_Abs_ to PhaP4_Abs_ models (QMEAN ranging from − 1.03 to − 3.60).

Due to the similarity between PhaP1_Abs_ and PhaP5_Reu_ (*R. eutropha* PhaP5) (55.8%) and based on the data that we obtained it was decided to focus on the analyses of the function of PhaP1_Abs_ on PHB granule morphology and PHB accumulation.

### PhaP1 does not affect growth of *A. brasilense*

**Fig. 3:**
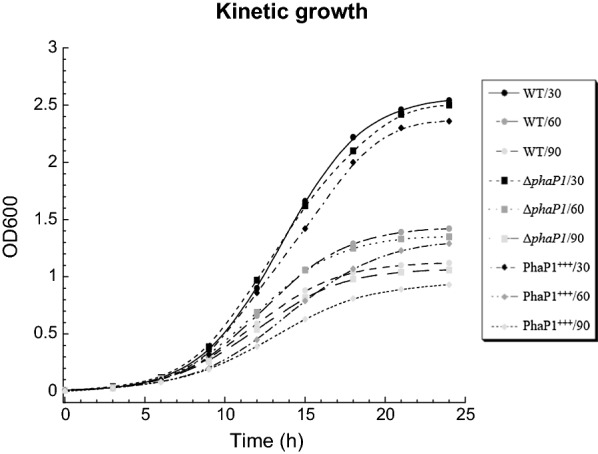
Growth kinetic behavior of *A. brasilense* strains. *A. brasilense* strains were inoculated in flasks containing minimal medium supplemented with malic acid and ammonium chloride, at C:N ratios of 30:1, 60:1 and 90:1, to an OD600 of 0.01. Samples were taken every 3 h for 24 h and measured spectrophotometrically at an OD600. Growth of *A. brasilense* Sp7 (WT) (circles), *A. brasilense* Δ*phaP1* (Δ*phaP1*) (squares) and *A. brasilense* PhaP1^+++^ (PhaP1^+++^) (diamonds). Dark spots indicate a C:N ratio of 30:1, gray spots for a C:N ratio of 60:1 and light gray spots denotes a C:N ratio of 90:1

Growth curves of *A. brasilense* strains were carried out using minimal medium containing malic acid and ammonium chloride at C:N ratios of 30:1, 60:1 and 90:1 (Fig. 3). At all evaluated C:N ratios, the exponential growth phase was reached at 15 h of culturing, whereas the stationary phase occurred at 21 h. As was expected, the strains of study grew better when a C:N ratio of 30:1 was used. By comparing the growth curves of the mutant (*A. brasilense* Δ*phaP1*) and complemented (*A. brasilense* PhaP1^+++^) strains with respect to the wild type (*A. brasilense* Sp7) strain was shown that PhaP1_Abs_ is not important for the growth of *A. brasilense*.

### Deletion of *phaP1* gene affects the morphology of the PHB granules

**Fig. 4:**
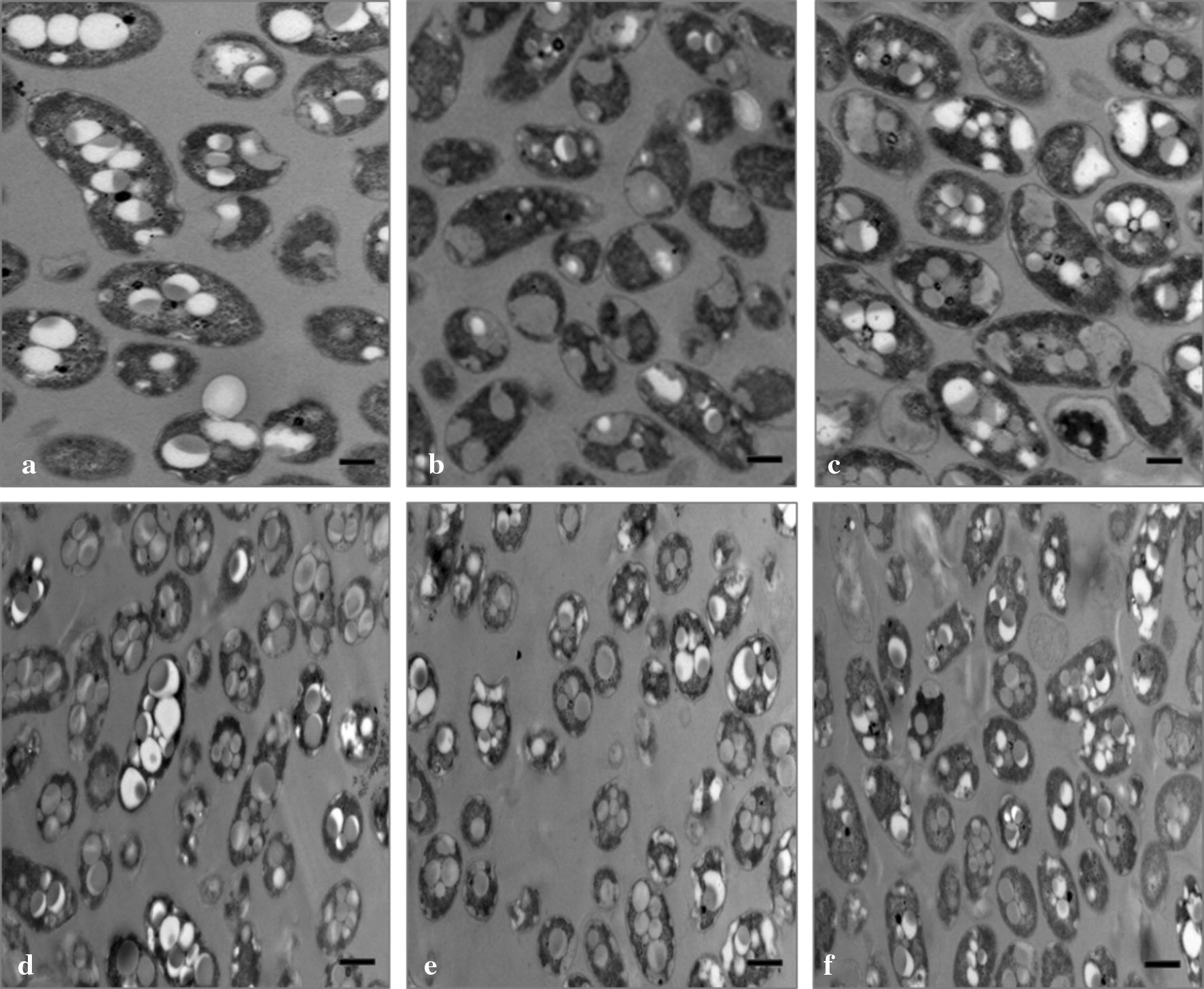
Transmission electron microscopy of ultra-thin sections of *A. brasilense.* Strains were grown on minimal medium containing malic acid and ammonium chloride at C:N ratios of 30:1 (**a**–**c**) and 90:1 (**e**–**g**). Strains were incubated at 32 °C and 110 rpm for 24 h. PHB granules of wild type (*A. brasilense* Sp7) (**a** and **e**), mutant (*A. brasilense* Δ*phaP1*) (**b** and **f**) and complemented (*A. brasilense* PhaP1^+++^) (**c** and **g**) strains are shown as intracellular granules. Label Scale bar = 1 µm

Wild type, mutant and complemented strains were cultivated for 15 h in minimal medium at C:N ratios of 30:1 and 90:1. Ultra-thin sections of the strains were analyzed by TEM (Fig. 4). At a C:N ratio of 30:1, several PHB granules were observed in the wild type strain. These granules are distributed throughout the cytoplasm (Fig. 4a). On the contrary, the mutant strain produced few PHB granules that appear to be larger and are located either at the center or at the pole of the cell (Fig. 4b). By restoring the PhaP1_Abs_ function in the complemented strain, the number and appearance of the PHB granules was restored (Fig. 4c). This suggests that at a C:N ratio of 30:1, PhaP1_Abs_ is involved in the control of the number and size of the PHB granules. However, when the bacteria were grown at a C:N ratio of 90:1, no differences in the size or distribution of the PHB granules could be observed (Fig. 4d–f). These results could suggest the participation of other PhaP proteins that contribute in the maintenance of the architecture of the PHB granules at the evaluated conditions.

### The deletion of the *phaP1* gene affects early (24 h) PHB accumulation depending on the C:N ratio used

**Fig. 5:**
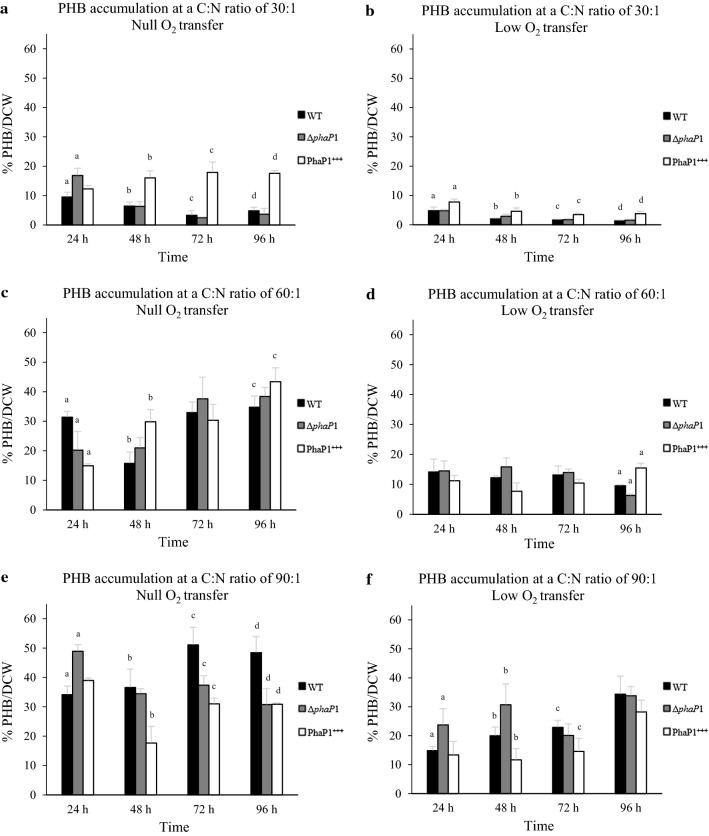
PHB/DCW content in *A. brasilense* strains. For PHB quantitation, flasks containing minimal medium supplemented with malic acid and ammonium chloride at C:N ratios of 30:1 (**a** and **b**), 60:1 (**c** and **d**), and 90:1 (**e** and **f**) were inoculated with the *A. brasilense* strains in flasks subjected to null (**a**, **c** and **e**) or low (**b**, **d** and **f**) oxygen transfer. Samples of cultures of *A. brasilense* were taken every 24 h for 96 h. Cells were harvested and dried, then PHB was extracted and quantified. PHB produced by *A. brasilense* Sp7 (WT), *A. brasilense* Δ*phaP1* (Δ*phaP1*) and *A. brasilense* PhaP1+++ (PhaP1^+++^) are shown. Data are presented as mean ± standard deviation of three independent experiments. Letters in each graphic denote significant differences (p > 0.05) according to a one-way ANOVA test

PHB accumulation was quantified to evaluate the possible involvement of PhaP1_Abs_ on biopolymer metabolism. Malic acid and ammonium chloride were used at C:N ratios of 30:1, 60:1 and 90:1 in cultures subjected to low or null oxygen transfer (as described in materials and methods). General comparison of the growth conditions evaluated for PHB accumulation (C:N ratio with low or null-oxygen transfer) is observed that the oxygen transfer limits PHB accumulation by approximately 50% (Fig. 5).

Moreover, by analyzing the PHB accumulation at a C:N ratio of 30:1 in cultures with null-oxygen transfer (Fig. 5a) it can be observed that the PHB is accumulated at 24 h of growth and subsequently degraded in wild type and mutant strain. However, it is observed that the mutant strain accumulates 17% PHB/DCW with respect to 9.5% PHB/DCW that was accumulated by the wild type strain. In addition, the PHB produced by the mutant is easily degraded to similar levels as wild type strain. This suggests that PhaP1_Abs_ somehow regulates the early stage of PHB accumulation. Moreover, the restoration of PhaP1_Abs_ function in the complemented strain results in an increased PHB content to reach 17.5% PHB/DCW, it is about 90% more PHB with respect to the PHB accumulated by wild type strain. It should be noted that there was no signal observed for PHB degradation, since the PHB content decreased with time in the wild type and mutant strains but it was increased in the complemented strain. We also quantified the PHB accumulation in *A. brasilense* strains cultured at a C:N ratio of 30:1 but subjected to low-oxygen transfer (Fig. 5b). Both, wild type and mutant strains reported a similar behavior in PHB accumulation. In these strains, PHB content reach the maximum level at 24 h (~ 5% PHB/DCW) and it was subsequently decreased to minimum levels (~ 1% PHB/DCW) at the end of bacterial culturing time (96 h). Taken together data obtained is suggested that PhaP1_Abs_ could be involved in the control of PHB synthesis or utilization at a C:N ratio of 30:1 in cultures subjected to null-oxygen transfer when early PHB synthesis occurs (24 h) (Fig. 5a). It should be noted that under low-oxygen transfer this does not occur (Fig. 5b).

To analyze whether the behavior observed is constant at other C:N ratios, it was decided to quantify PHB accumulation by increasing the C:N ratio to 60:1 and 90:1 under low or null-oxygen transfer (Fig. 5c–f). The data obtained at a C:N ratio of 60:1 in cultures with null-oxygen transfer showed a similar behavior by wild type, mutant and complemented strains from 72 to 96 h. However, at 24 h it appears that PHB synthesis in the mutant and complemented strains is delayed but at 48 h, they accumulated more PHB than the wild type strain (Fig. 5c). The observed phenotype may be due to the presence of another phasin protein that support PHB accumulation when a C:N ratio of 60:1 is combined with null-oxygen transfer. The above mentioned does not occur when PHB is measured in cultures subjected to low-oxygen transfer as can be observed in Fig. 5d.

Furthermore, when the PHB was quantified in cultures grown at a C:N ratio of 90:1 was observed that PHB is rapidly synthesized by the mutant strain when it is grown in cultures subjected to a null-oxygen transfer. At 24 h, the mutant strain accumulates approximately 50% PHB/DCW, whereas the wild type strain accumulates 35% PHB/DCW (Fig. 5e). It should be noted that the excess of PHB accumulated by the mutant strain is degraded after 48 h and remains constant up to 96 h of culture. Whereas, the PHB produced by the wild type strain increases over time. The complemented strain does not recover the wild type phenotype in PHB accumulation (Fig. 5e). Moreover, when cultures were grown at a C:N ratio of 90:1 and subjected to a low-oxygen transfer (Fig. 5f), it was found that, between 24 and 48 h of culture, the mutant strain increases the PHB content from 50 to 60% with respect to the wild type strain. PHB is accumulated rapidly by the mutant strain in contrast to a slow accumulation of PHB by the wild type.

### Differential transcription of putative phasin genes under three different C:N ratios

**Fig. 6:**
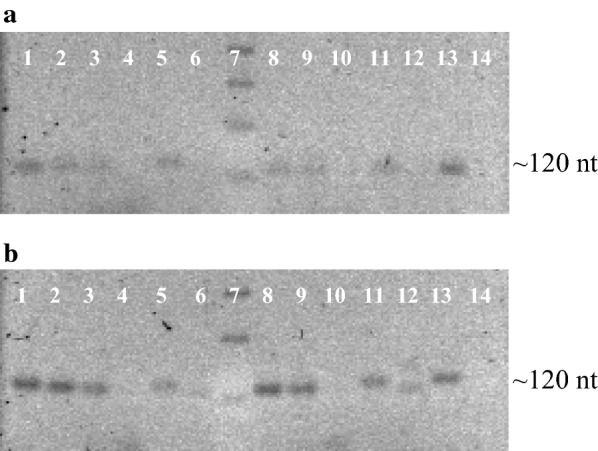
Analysis of the transcription of *A. brasilense* Sp7 phasin genes. RT-PCR assays of the internal region of putative phasin genes were conducted using cDNA of wild type (*A. brasilense* Sp7) and mutant (*A. brasilense* Δ*phaP1*) strains. RNA was extracted from cultures grown on minimal medium containing malic acid and ammonium chloride at C:N ratios of 30:1 and 90:1. Transcripts were visualized on a 3% agarose gel electrophoresis. **a** Transcription analyses of putative phasin genes of wild type and mutant strains grown at a C:N ratio of 30:1. For wild type: *phaP1* (lane 1), *phaP2* (lane 2), *phaP3* (lane 3), *phaP4* (lane 4), *phaP5* (lane 5), *phaP6* (lane 6). GeneRuler 1 Kb DNA ladder ThermoScientific (lane 7). For mutant strain: *phaP2* (lane 8), *phaP3* (lane 9), *phaP4* (lane 10), *phaP5* (lane 11), *phaP6* (lane 12). 16S rRNA was used as positive control (lane 13). Negative control using cDNA of mutant strain with gene-specified primers for *phaP1* transcript was done (lane 14). **b** Transcription analyses of putative phasin genes in wild type and mutant strains grown at a C:N ratio of 90:1. For wild type strain: *phaP1* (lane 1), *phaP2* (lane 2), *phaP3* (lane 3), *phaP4* (lane 4), *phaP5* (lane 5), *phaP6* (lane 6). GeneRuler 1 Kb DNA ladder ThermoScientific (lane 7). For mutant strain: *phaP2* (lane 8), *phaP3* (lane 9), *phaP4* (lane 10), *phaP5* (lane 11), *phaP6* (lane 12). 16S rRNA was used as positive control (lane 13). Negative control using cDNA of mutant strain with gene-specified primers for *phaP1* transcript was done (lane 14)

TEM images obtained from ultra-thin sections of *A. brasilense* strains grown at a C:N ratio of 90:1 showed no differences in the size and number of PHB granules by both, mutant and complemented strains, compared to the wild type strain (Fig. 4d–f). Additionally, the data obtained from the PHB quantification at C:N ratios of 60:1 and 90:1 showed an unclear phenotype. This suggests the possible involvement of other phasin proteins for the PHB accumulation and the maintenance of the structure of the PHB granules. To address this possibility, the transcripts of the *phaP* genes of wild type and mutant strains were evaluated by RT-PCR (Fig. 6). The results show that, *phaP1* and *phaP2* are the main genes transcribed in the wild type strain when cultures are grown at a C:N ratio of 30:1 (Fig. 6a). However, in the mutant, the *phaP2* transcript was the most prominent of all phasins and the *phaP3* transcript was partially increased (Fig. 6a). Nevertheless, it is still possible that the *phaP2* product could compensate for the lack of PhaP1_Abs_ in the granule structure as well as the PHB content in the mutant strain. Transcripts of the *phaP4* and *phaP6* were not observed either in wild type and mutant strains and the transcript of *phaP5* was slightly detected at a C:N ratio of 30:1 (Fig. 6a). However, when *A. brasilense* was grown at a C:N ratio of 90:1 (Fig. 6b), *phaP3* transcript increased to levels similar to *phaP2* in the wild type strain. A similar behavior was observed for the mutant strain (Fig. 6b). It should be mentioned that the transcript of the *phaP4* gene was not observed in both, wild type and mutant strains, whereas the *phaP5* and *phaP6* transcripts were present at low levels (Fig. 6b). Our results suggest that the C:N ratio has important implications on phasin genes transcription in this bacterium.

## Discussion

The function of PhaP1_Abs_ on PHB synthesis and PHB granule structure in *A. brasilense* was analyzed. Phasin proteins have been mainly implicated in PHB granule stabilization which occurs due to their amphiphilic properties (Pötter and Steinbüchel 2005) that avoid the coalescence of the PHB granules (Wieczorek et al. 1995; Pfeiffer et al. 2011). Every PHB-producing microorganism has at least one phasin, however there are reports of bacteria with several phasin proteins such as *R. eutropha* with seven PhaP proteins (Wieczorek et al. 1995; Pötter et al. 2004; Pfeiffer and Jendrossek 2011, 2012). Despite the existence of multiple homologues in some bacterial species and the fact that most of them can be bound to the PHB granule, only one or two are covering the PHB granules (Pötter et al. 2004; Yoshida et al. 2013). In this study, bioinformatic analyses of the *A. brasilense* Sp7 genome revealed six putative phasin genes, referred as *phaP1* to *phaP6*. The amino acid sequences of *A. brasilense* phasins are not highly conserved however similar results have been reported for phasins of *R. eutropha* (Pötter et al. 2004; Pfeiffer and Jendrossek 2012), *H. seropedicae* (Alves et al. 2016), *Bradyrhizobium japonicum* (Yoshida et al. 2013) and *Haloferax mediterranei* (Cai et al. 2012) among others. The predicted secondary structures of PhaP1_Abs_ to PhaP5_Abs_ reveals a high percentage of α-helix content as has been reported for this type of proteins (Mezzina and Pettinari 2016; Zhao et al. 2016).

The deletion of the main phasin that covers the PHB granules results in a single enlarged granule rather than five to ten granules that are observed in the wild type strains (Steinbüchel et al. 1995; Jurasek and Marchessault 2002; Cai et al. 2012). To analyze the role of the PhaP1_Abs_ in the PHB granule morphology, the *phaP1* gene was deleted. TEM images of the carbonosomes from the mutant strain showed fewer PHB granules that were larger than those observed in the wild type strain. This strongly suggests that PhaP1_Abs_ controls to a certain extent the number and size of the PHB granules.

Among other functions attributable to phasins are the regulation of synthesis and degradation of PHB (York et al. 2001; Handrick et al. 2004a, b; Cai et al. 2012) and the distribution of the PHB granules during cell division (Galán et al. 2011; Pfeiffer and Jendrossek 2011; Wahl et al. 2012; Bresan and Jendrossek 2017). To analyze the possible involvement of PhaP1_Abs_ on PHB synthesis and degradation in *A. brasilense* Sp7, a *phaP1* mutant strain was constructed and PHB accumulation was determined. For that, minimal medium supplemented with malic acid and ammonium chloride at different C:N ratios in addition to either low or null-oxygen transfer conditions was used. The results showed that PhaP1_Abs_ controls early PHB synthesis at a C:N ratio of 30:1 when the oxygen transfer is null. The overexpression of PhaP1_Abs_ in the complemented strain resulted in the increase of PHB over time, suggesting a possible increment in the PHB synthesis or a decrement in the PHB utilization. However, when the accumulation of PHB in *A. brasilense* was quantified in cultures grown in minimal medium at C:N ratios of 60:1 and 90:1 and subjected to low or null-oxygen transfer, no clear effect caused by the lack or overexpression of PhaP1_Abs_ was observed. This suggest a possible participation of other phasin proteins to maintain PHB accumulation.

By analyzing the behavior of phasins in *R. eutropha*, it was found that the *phaP1* mutant strain accumulates approximately 40% PHB/DCW less than the wild type strain (Pötter et al. 2005). However, the deletion of *phaP2*, *phaP3* or *phaP4* did not affect PHB accumulation (Pötter et al. 2005). In *H. seropedicae* SmR1, the deletion of *phaP1* reduced PHB accumulation by 50% PHB/DCW but the *pha*P2 deleted strain produced 30% PHB/DCW more than the wild type strain when grown on glucose as a carbon source, suggesting that PhaP2_Hse_ (*Herbaspirillum seropedicae* PhaP2) compensates for the lack of PhaP1_Hse_ (Alves et al. 2016). Similar results have been obtained for *Methylobacterium extorquens* AM1 (Korotkova et al. 2002). The deletion of *phaP1* and *phaP2* in *H. seropedicae* and *phaP1* to *phaP4* in *R. eutropha* H16 abolished PHB accumulation (Pötter et al. 2005; Alves et al. 2016). These results, in addition to the changes observed in polymer accumulation allow to infer a possible interaction between phasin proteins and the PHB polymerase, which has been corroborated for *R. eutropha* (York et al. 2001), *Aeromonas caviae* (Fukui et al. 2001; Ushimaru et al. 2014) *A. hydrophila* (Tian et al. 2005), and, in this work, is suggested for *A. brasilense* Sp7, however the mechanism by which the interactions take place has not been elucidated.

Furthermore, a strong interaction between PhaP5_Reu_ and the PHB depolymerase or the regulator protein (PhaR) has been proven (Pötter et al. 2005; Pfeiffer and Jendrossek 2011). Studies of PhaP2_Reu_ and PhaP4_Reu_ on PHB degradation conclude that both phasins, limit PHB degradation by interacting with the depolymerases PhaZ2, PhaZ3 and PhaZ7. Moreover, the expression of PhaP1_Reu_ reduced the PHB content in *E. coli* when expressing the *phaCAB* operon of *R. eutropha* (Eggers and Steinbüchel 2014).

In conclusion, in this study we demonstrated that early PHB synthesis and the morphology of the PHB granules are influenced by PhaP1_Abs_ when *A. brasilense* Sp7 is grown in a medium with a C:N ratio of 30:1. Nevertheless, when C:N ratios of 60:1 and 90:1 were used, PHB synthesis and the morphology of the PHB granules seem to be influenced by the coordinated action of several phasin proteins.

## Supplementary information

**Additional file 1.** Putative phasin genes localization.

**Additional file 2.** Identity and similarity percentages between the entire amino acids sequences of phasins from PHB-producing microorganisms.

**Additional file 3.** Ramachandran plots of predicted three-dimensional structures of putative *A. brasilense* Sp7 phasins.

## Data Availability

Please contact to the authors for all request.

## Abbreviations

PHB: polyhydroxybutyrate
[P(HB-*co*-HHx)]: poly(3-hydroxybutyrate-*co*-3-hydroxyhexanoate)
GAP’s: granule associated proteins
C:N: carbon:nitrogen ratio
LB: Luria–Bertani
h: hours
min: minutes
s: seconds
Kg: kilograms
g: grams
*g*: gravity
OD600: optical density at 600 nm
DCW: dry cell weight
TEM: transmission electron microscopy
