# Supplementary figures and images for: Phasin PhaP1 is involved in polyhydroxybutyrate granules morphology and in controlling early biopolymer accumulation in Azospirillum brasilense Sp7
Source: AMB Express. 2019 Sep 25;9:155. doi: 10.1186/s13568-019-0876-4 (PMC6761214; doi:10.1186/s13568-019-0876-4)

**Putative phasin genes localization**


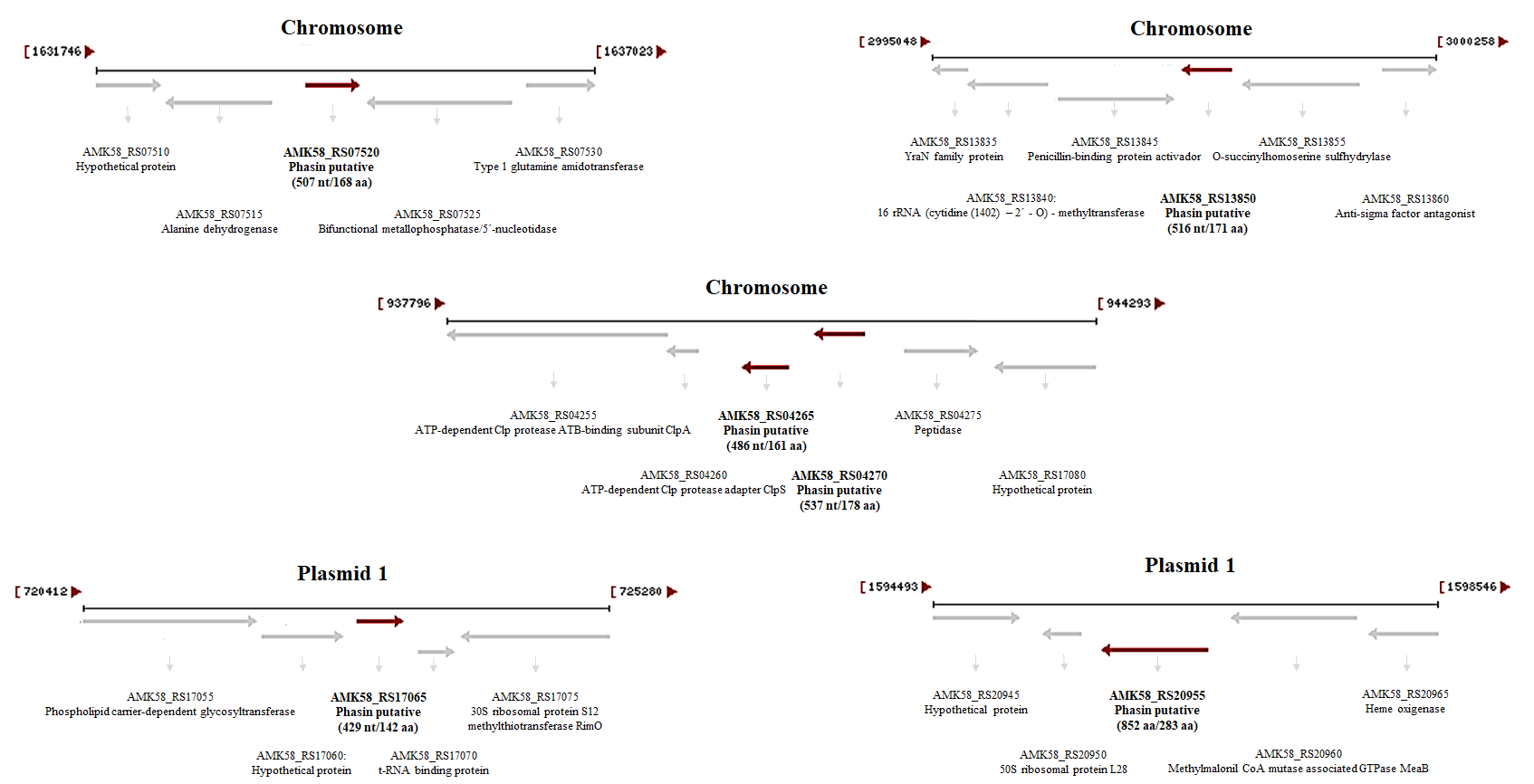


nt: nucleotides; aa: amino acids

Supplement: Supplementary file 1 — Additional file 1. Putative phasin genes localization. [file 13568_2019_876_MOESM1_ESM.docx]
