# Supplementary material for: Phasin PhaP1 is involved in polyhydroxybutyrate granules morphology and in controlling early biopolymer accumulation in Azospirillum brasilense Sp7
Source: AMB Express. 2019 Sep 25;9:155. doi: 10.1186/s13568-019-0876-4 (PMC6761214; doi:10.1186/s13568-019-0876-4)
Supplement: Supplementary file 2 — Additional file 2. Identity and similarity percentages between the entire amino acids sequences of phasins from PHB-producing microorganisms. [file 13568_2019_876_MOESM2_ESM.docx]

**Identity and similarity percentages between the entire amino acids sequences of phasins from PHB-producing microorganisms^1^.**

| **SIMILARITY (%)** | | | | | | | | | | | | | | | | | | | | | |
| --- | --- | --- | --- | --- | --- | --- | --- | --- | --- | --- | --- | --- | --- | --- | --- | --- | --- | --- | --- | --- | --- |
| **IDENTITY (%)** |  | **PhaP1**  **Abs** | **PhaP2**  **Abs** | **PhaP3**  **Abs** | **PhaP4**  **Abs** | **PhaP5**  **Abs** | **PhaP6**  **Abs** | **PhaP1**  **Reu** | **PhaP2**  **Reu** | **PhaP3**  **Reu** | **PhaP4**  **Reu** | **PhaP5**  **Reu** | **PhaP6**  **Reu** | **PhaP7**  **Reu** | **PhaM**  **Reu** | **PhaF**  **Ppu** | **PhaI**  **Ppu** | **PhaP**  **AFA8** | **PhaP1**  **Hse** | **PhaP2**  **Hse** | **PhaP**  **Ahy** |
|  | **PhaP1**  **Abs** |  | 37.3 | 34.9 | 26.0 | 21.2 | 18.8 | 31.1 | 24.7 | 29.4 | 25.5 | 55.8 | 18.6 | 27.6 | 14.6 | 11.6 | 16.4 | 27.2 | 25.7 | 28.3 | 27.5 |
|  | **PhaP2**  **Abs** | 14.9 |  | 60.7 | 38.7 | 26.9 | 18.4 | 24.5 | 20.1 | 21.4 | 20.6 | 26.5 | 21.1 | 18.6 | 14.9 | 10.7 | 14.8 | 23.0 | 24.6 | 24.8 | 22.2 |
|  | **PhaP3**  **Abs** | 15.8 | 42.5 |  | 36.7 | 28.9 | 17.3 | 22.2 | 16.1 | 18.0 | 16.5 | 24.8 | 20.1 | 15.5 | 10.5 | 10.7 | 14.7 | 20.7 | 20.2 | 20.0 | 20.3 |
|  | **PhaP4**  **Abs** | 11.5 | 18.3 | 17.1 |  | 24.7 | 15.7 | 17.4 | 15.4 | 15.2 | 13.4 | 23.8 | 23.6 | 14.8 | 10.5 | 8.0 | 11.7 | 15.8 | 16.6 | 14.1 | 21.7 |
|  | **PhaP5**  **Abs** | 9.8 | 12.4 | 14.2 | 13.0 |  | 12.2 | 15.2 | 15.7 | 17.0 | 17.0 | 22.9 | 33.4 | 16.1 | 13.0 | 8.0 | 11.7 | 13.0 | 15.9 | 18.2 | 15.7 |
|  | **PhaP6**  **Abs** | 7.8 | 7.9 | 7.2 | 6.4 | 5.3 |  | 20.9 | 20.2 | 16.9 | 20.2 | 17.2 | 10.0 | 12.0 | 24.3 | 23.5 | 7.7 | 18.1 | 19.3 | 19.0 | 12.7 |
|  | **PhaP1**  **Reu** | 12.5 | 10.7 | 11.5 | 5.6 | 5.6 | 10.4 |  | 57.7 | 66.1 | 60.8 | 25.9 | 12.8 | 24.1 | 26.4 | 17.6 | 12.9 | 55.9 | 59.1 | 61.2 | 18.9 |
|  | **PhaP2**  **Reu** | 9.5 | 7.8 | 6.0 | 5.7 | 6.8 | 9.4 | 40.7 |  | 54.5 | 78.8 | 20.1 | 11.3 | 21.3 | 22.9 | 14.5 | 11.8 | 48.6 | 46.1 | 47.6 | 19.8 |
|  | **PhaP3**  **Reu** | 11.5 | 8.9 | 8.6 | 5.8 | 5.4 | 8.5 | 47.4 | 34.9 |  | 56.0 | 24.5 | 13.3 | 26.1 | 25.4 | 14.0 | 12.5 | 46.5 | 57.9 | 53.9 | 19.1 |
|  | **PhaP4**  **Reu** | 10.4 | 8.3 | 8.8 | 4.6 | 6.0 | 9.7 | 43.3 | 68.7 | 36.5 |  | 22.1 | 13.3 | 22.7 | 22.5 | 14.2 | 11.8 | 49.2 | 47.1 | 47.6 | 16.6 |
|  | **PhaP5**  **Reu** | 33.7 | 10.4 | 10.8 | 9.4 | 10.4 | 5.8 | 13.2 | 9.1 | 8.8 | 9.1 |  | 18.1 | 22.7 | 17.8 | 11.1 | 14.8 | 23.7 | 25.6 | 26.3 | 24.3 |
|  | **PhaP6**  **Reu** | 7.2 | 8.8 | 7.9 | 9.4 | 20.6 | 3.6 | 4.4 | 3.4 | 3.1 | 4.1 | 10.5 |  | 12.9 | 8.8 | 6.7 | 9.5 | 12.4 | 13.1 | 13.0 | 11.1 |
|  | **PhaP7**  **Reu** | 9.8 | 6.0 | 4.5 | 6.7 | 5.8 | 6.3 | 10.4 | 7.7 | 11.3 | 9.6 | 9.8 | 3.6 |  | 13.0 | 8.5 | 13.1 | 22.9 | 21.8 | 21.1 | 20.8 |
|  | **PhaM**  **Reu** | 4.5 | 5.3 | 3.7 | 5.4 | 5.4 | 13.0 | 13.5 | 12.8 | 12.8 | 13.5 | 8.3 | 2.4 | 6.6 |  | 24.8 | 8.0 | 20.9 | 23.3 | 24.7 | 10.6 |
|  | **PhaF**  **Ppu** | 4.1 | 4.5 | 4.8 | 3.3 | 3.0 | 14.7 | 10.0 | 7.6 | 8.5 | 7.8 | 3.9 | 2.9 | 2.6 | 15.3 |  | 26.1 | 16.0 | 18.2 | 17.3 | 8.7 |
|  | **PhaI**  **Ppu** | 4.6 | 6.0 | 7.3 | 5.8 | 4.0 | 1.4 | 4.8 | 4.5 | 3.3 | 4.9 | 5.6 | 4.5 | 4.6 | 4.1 | 17.4 |  | 10.7 | 15.8 | 14.0 | 14.9 |
|  | **PhaP**  **AFA8** | 8.6 | 12.8 | 8.9 | 7.3 | 3.6 | 7.4 | 35.9 | 29.8 | 27.8 | 28.8 | 8.2 | 5.8 | 9.7 | 11.5 | 10.2 | 4.5 |  | 49.2 | 45.8 | 20.1 |
|  | **PhaP1**  **Hse** | 11.4 | 11.8 | 11.3 | 5.4 | 6.1 | 10.3 | 42.3 | 30.0 | 39.8 | 30.5 | 13.5 | 4.8 | 10.6 | 10.1 | 9.4 | 6.6 | 32.4 |  | 69.3 | 21.8 |
|  | **PhaP2**  **Hse** | 11.7 | 12.2 | 8.4 | 4.2 | 6.0 | 8.0 | 46.4 | 31.0 | 33.3 | 30.0 | 12.9 | 4.7 | 9.1 | 13.2 | 9.1 | 5.7 | 27.6 | 58.8 |  | 24.2 |
|  | **PhaP**  **Ahy** | 10.9 | 6.8 | 5.7 | 7.9 | 3.6 | 4.8 | 5.9 | 8.1 | 6.7 | 6.5 | 10.2 | 2.9 | 10.0 | 4.7 | 2.1 | 2.8 | 10.8 | 9.9 | 8.7 |  |

^1^ PhaP1Abs to PhaP6Abs denote *A. brasilense* Sp7 PhaP1 to PhaP6. PhaP1Reu to PhaP7Reu and PhaMReu indicate *R.eutropha* PhaP1 to PhaP7 and PhaM. PhaFPpu and PhaIPu refers to *P. putida* PhaI and PhaF. PhaPAFA8 denotes *Azotobacter* FA8. PhaP1Hse and PhaP2Hse refer to *H. seropedicae* PhaP1 and PhaP2. PhaPAhy indicates *A. hydrophila* PhaP.
