# Supplementary material for: Phasin PhaP1 is involved in polyhydroxybutyrate granules morphology and in controlling early biopolymer accumulation in Azospirillum brasilense Sp7
Source: AMB Express. 2019 Sep 25;9:155. doi: 10.1186/s13568-019-0876-4 (PMC6761214; doi:10.1186/s13568-019-0876-4)
Supplement: Supplementary file 3 — Additional file 3. Ramachandran plots of predicted three-dimensional structures of putative A. brasilense Sp7 phasins. [file 13568_2019_876_MOESM3_ESM.docx]

**Ramachandran plots of predicted three-dimentional structures of putative *A. brasilense* Sp7 phasins**


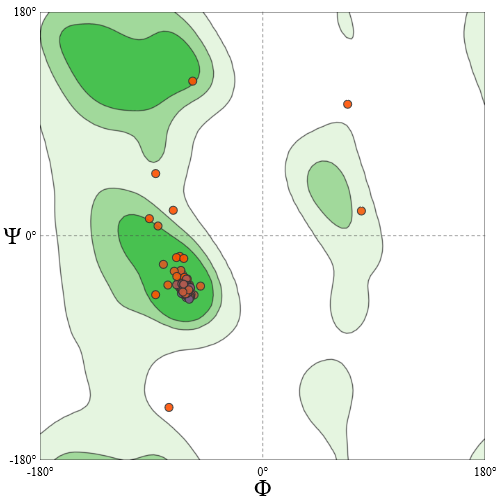


a) PhaP1_Abs_


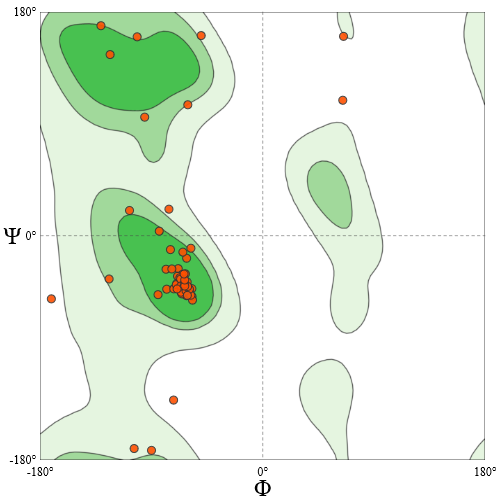


b) PhaP2_Abs_


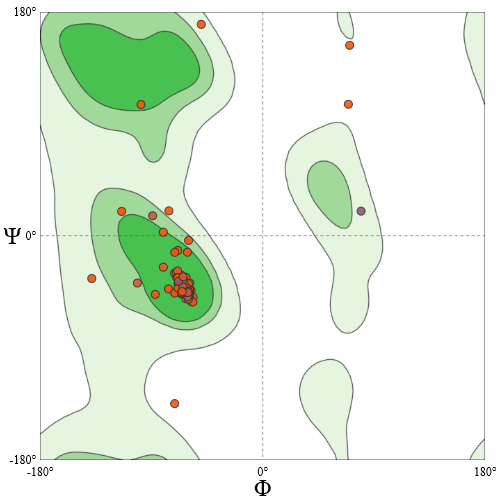


c) PhaP3_Abs_


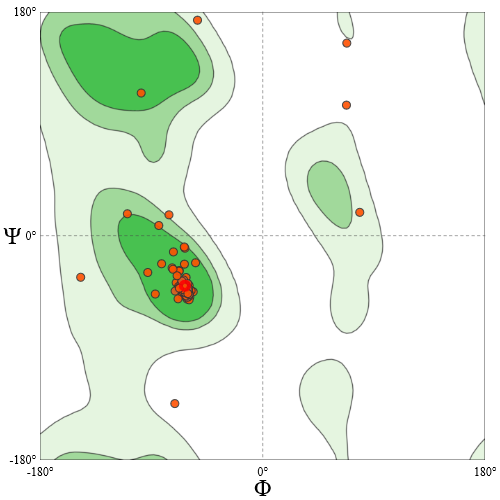


d) PhaP4_Abs_


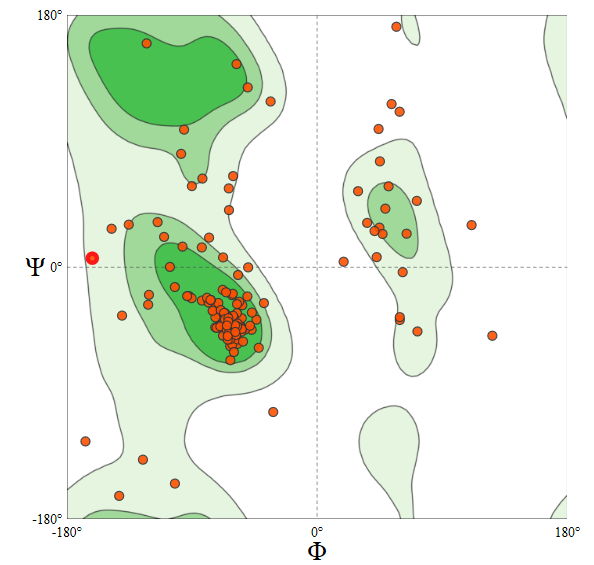


e) PhaP5_Abs_


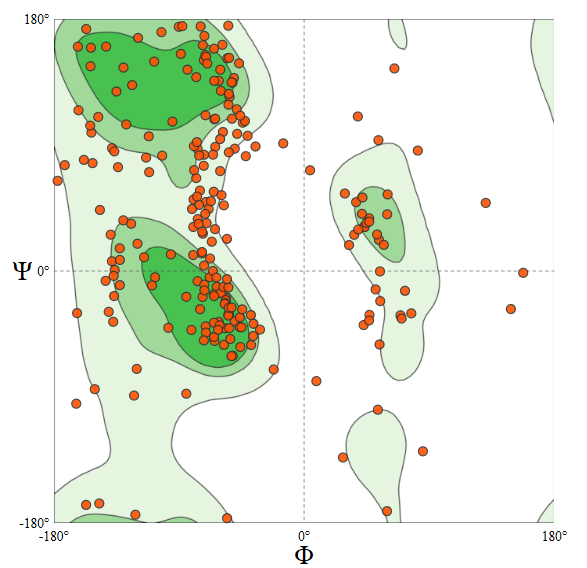


f) PhaP6_Abs_
